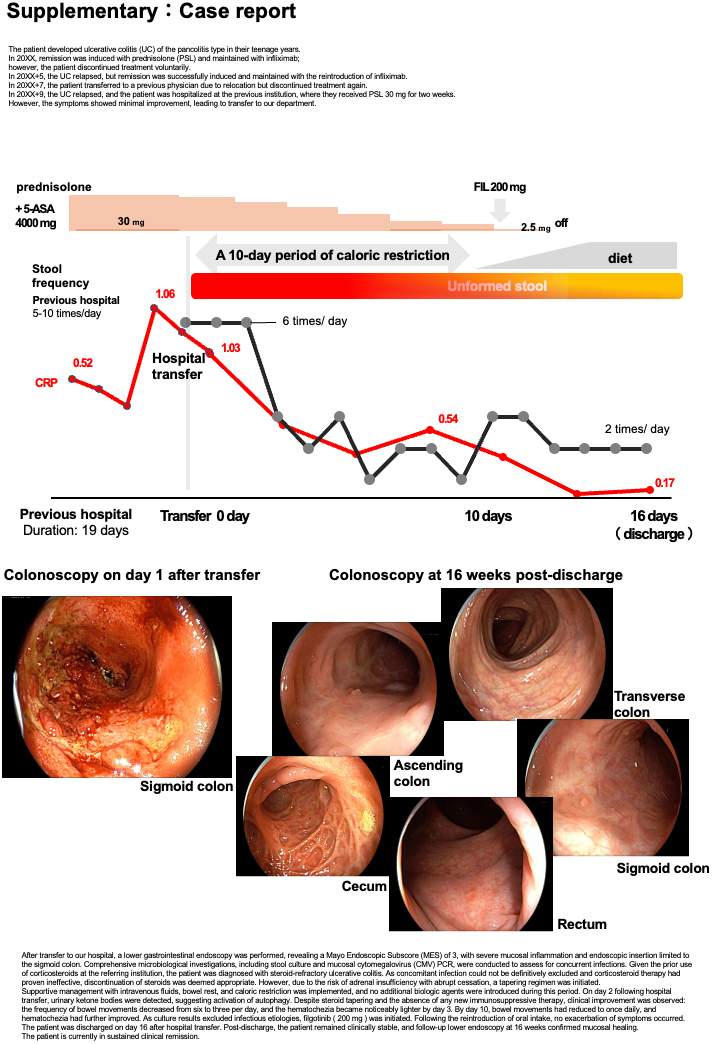# Caloric Restriction Is Associated With Enhanced Clinical Outcomes in Hospitalized Patients With Ulcerative Colitis

**DOI:** 10.1016/j.gastha.2025.100842

**Published:** 2025-11-07

**Authors:** Tomoyuki Nagai, Yoriaki Komeda, Saki Yoshida, Kohei Handa, Sho Masaki, Masashi Kono, Hajime Honjo, Shigenaga Matsui, Naoko Tsuji, Hiroshi Kashida, Masatoshi Kudo

**Affiliations:** Department of Gastroenterology and Hepatology, Kindai University Faculty of Medicine, Osaka, Japan

**Keywords:** Autophagy, Caloric restriction, Ulcerative colitis

## Abstract

**Background and Aims:**

Ulcerative colitis (UC) is a chronic inflammatory bowel disease with a relapsing-remitting course that often requires hospitalization during flares. While prolonged fasting has traditionally led to high-calorie intravenous nutrition, excessive caloric intake may induce hyperglycemia, increase infection risk, and inhibit autophagy. This study aimed to evaluate the impact of a short-term, calorie-restricted regimen (≤400 kcal/day), designed to promote autophagy, on clinical outcomes in hospitalized patients with UC.

**Methods:**

A retrospective analysis was conducted on 38 patients admitted for UC exacerbation between January 2022 and December 2024. Patients were categorized into a calorie-restricted group (≤400 kcal/day) or a standard nutrition group (>400 kcal/day) based on their total caloric intake. The calorie-restricted group received only intravenous fluids and noncaloric beverages. The primary endpoint was clinical remission at day 14, while secondary endpoints included the length of hospital stay and incidence of adverse events.

**Results:**

The clinical remission at day 14 following treatment initiation was significantly higher in the calorie-restricted group (86% [12/14]) compared to the standard nutrition group (42% [10/24]) (*P* < .05). The mean duration of hospitalization was also significantly shorter in the calorie-restricted group (11.0 ± 3.3 days) compared to the standard nutrition group (22.1 ± 8.9 days) (*P* < .01). The calorie-restricted group experienced mild, transient adverse events but no serious complications. In contrast, the standard nutrition group experienced serious adverse events such as catheter-related infections and myocarditis.

**Conclusion:**

Caloric restriction during hospitalization for UC exacerbation may be associated with increased clinical remission and a shorter hospital stay. That nutritional strategy offers a potentially novel and cost-effective approach distinct from conventional bowel rest. Further prospective, multicenter studies are warranted to validate these findings.

## Graphical abstract

**Figure:**
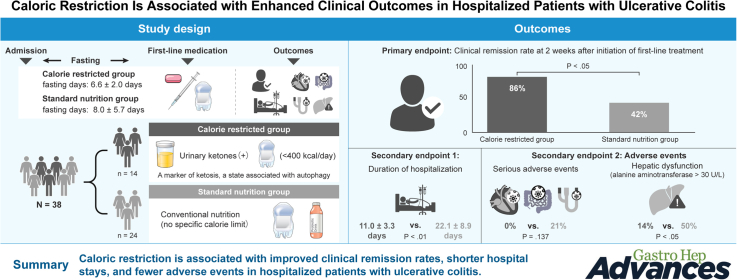

## Introduction

Ulcerative colitis (UC) is a chronic inflammatory bowel disease (IBD) characterized by recurrent flares and periods of remission, often requiring hospitalization.^1^ Standard therapeutic approaches for UC include the use of 5-aminosalicylic acid (5-ASA), corticosteroids, and immunomodulators (azathioprine, 6-mercaptopurine). In addition, biologic agents, such as tumor necrosis factor-α inhibitors, integrin inhibitors, and Janus kinase inhibitors, have been introduced as effective treatment modalities.^1^ However, an optimal approach for intravenous fluid and nutritional management in hospitalized patients with UC exacerbation is yet to be established.

Traditionally, the management of hospitalized patients with UC has emphasized “intestinal rest,” wherein energy requirements are estimated using predictive equations, such as the Harris–Benedict formula, and nutritional support is provided via parenteral nutrition (PN).^2^^,^^3^ However, the clinical benefits of the intestinal rest remain unclear. Park et al. reported no significant differences in disease activity improvement between the fasting and oral feeding groups among hospitalized patients with IBD.^4^ Consequently, recent clinical guidelines recommend avoiding unnecessary fasting in patients who are capable of oral intake.^5^

Recent evidence has raised concerns about the metabolic and immunological consequences of high-calorie PN. PN induces hyperglycemia and hyperinsulinemia, increasing the risk of catheter-related bloodstream infection and thrombosis.^6^^,^^7^ Furthermore, the Early Parenteral Nutrition in Critically Ill patients (EPaNIC) study demonstrated that early PN administration in critically ill patients is linked to poor clinical outcomes, potentially due to suppression of autophagy.^6^ study demonstrated that early PN administration in critically ill patients is linked to poor clinical outcomes, potentially due to suppression of autophagy.^6^ Autophagy is a fundamental cellular process that maintains homeostasis through the degradation of damaged organelles and misfolded proteins. It also plays a crucial role in immune regulation and inflammatory control.^8^

It has been hypothesized that nutritional overloading may suppress autophagy, thereby impairing intestinal epithelial repair mechanisms. *The* PRotEin provision in Critical IllneSs (PRECISe study) suggested that excessive protein intake through enteral nutrition (EN) might exacerbate intestinal inflammation,^9^ highlighting the need to reconsider current nutritional strategies. Low-calorie dietary regimens may promote autophagy and contribute to inflammation modulation.^6^^,^^8^, ^9^, ^10^ Although the precise role of autophagy in the pathogenesis of IBD remains unclear, genetic studies have identified autophagy-related genes (*immunity-related GTPase M, LRRK2, SMURF1, and ATG16L1*) as the susceptibility loci for IBD, further supporting the hypothesis that autophagy plays a pivotal role in UC pathophysiology.^11^

Despite increasing evidence suggesting that autophagy modulation has therapeutic potential, nutritional interventions aimed at inducing autophagy in UC have not yet been systematically explored. Although caloric restriction has been implicated in the modulation of various disease states beyond simple energy regulation, its clinical application in UC remains limited.^10^

In this study, we aimed to investigate the clinical efficacy of caloric restriction (≤400 kcal/d) as a strategy to induce autophagy, rather than fasting solely for intestinal rest. The safety of this approach is supported by the results of previous studies that utilized low-calorie ketogenic diets in patients with psoriasis, where no significant adverse effects were reported.^12^

At our institution, we have implemented a carefully monitored nutritional management protocol for hospitalized patients with UC, with particular attention paid to potential vitamin and mineral deficiencies and the risk of refeeding syndrome. In this retrospective analysis, we evaluated whether caloric restriction could enhance remission induction rates and reduce the duration of hospitalization in patients with UC. Given the emerging evidence suggesting that autophagy plays a key role in immune regulation and resolution of inflammation, the findings of this study may provide novel insights into the metabolic regulation of UC and contribute to the redefinition of nutritional strategies for hospitalized patients.

To illustrate the clinical relevance of this approach, we present a representative case of a young patient with pancolitis-type UC who previously achieved remission with infliximab but experienced multiple relapses after treatment discontinuation. Upon hospitalization at another institution, the patient received prednisolone (30 mg) for 2 weeks with minimal improvement. Following transfer to our department, endoscopic evaluation confirmed active mucosal inflammation, and the patient was diagnosed with steroid resistance. The clinical course of this patient is detailed in the Supplementary Case report.

Instead of escalating to biologics immediately, we initiated a carefully monitored caloric restriction protocol (<400 kcal/d), along with intravenous hydration. Within 48 hours, urinary ketone bodies were detected, suggesting the activation of autophagy. The patient’s stool frequency improved from six times/day to two times/day, and systemic inflammation, as measured by C-reactive protein (CRP), showed a decreasing trend. The patient achieved clinical remission without requiring second-line biologics and was discharged after a hospital stay of 16 days, which was shorter than the 19 days spent without improvement at the previous hospital. This case highlights the potential of caloric restriction therapy to facilitate remission induction, even in biologically experienced patients with steroid-resistant UC.

### Patients

This retrospective cohort study included patients with UC who were hospitalized for disease management at Kindai Hospital between January 2022 and December 2024. Patients were eligible for inclusion if they met all of the following criteria: (1) confirmed diagnosis of UC according to the criteria established by the Research Group for Intractable IBD of the Ministry of Health, Labour and Welfare of Japan; (2) hospitalization due to disease exacerbation; (3) receipt of corticosteroids, tacrolimus, infliximab, other biological agents, or PN during admission; (4) age ≥15 years; (5) for patients with multiple hospitalizations, only the first admission during the study period being considered; and (6) not requiring exclusion for the presence or absence of 5-ASA allergy.

The exclusion criteria were as follows: (1) diagnosis of other types of colitis (eg, infectious colitis or ischemic colitis), (2) a history of colectomy prior to admission, (3) presence of bloody stools due to diverticular bleeding, and (4) participation in clinical trials involving investigational drugs.

Eligible patients were categorized into 2 groups according to their caloric intake during hospitalization: the calorie-restricted group (≤400 kcal/d) and the standard nutrition group (>400 kcal/d). Both groups were allowed to drink fluids; however, the calorie-restricted group was limited to noncaloric beverages, specifically water and unsweetened tea.

## Materials and Methods

This single-center retrospective cohort study was conducted at Kindai University Hospital to evaluate the efficacy and safety of caloric restriction in hospitalized patients with UC. Clinical data were extracted from electronic medical records and included vital signs, stool frequency, stool consistency, disease severity (Partial Mayo Score [PMS]), endoscopic findings (Mayo Endoscopic Subscore [MES]), laboratory markers (CRP, hemoglobin, albumin [Alb]), physician-directed dietary interventions, nutritional status, intravenous fluid composition, and treatment regimens.

The caloric intake for all patients was assessed based on both intravenous fluid composition and physician-directed dietary interventions. The calorie-restricted group (≤400 kcal/day) was provided with intravenous fluids and noncaloric beverages such as water and unsweetened tea. No EN was used in either group during the fasting period. The intravenous fluid content for the calorie-restricted group was primarily comprised of low-calorie maintenance solutions focused on correcting electrolyte imbalances and supplementing vitamins, based on daily blood test results. This regimen was designed to deplete hepatic glycogen and induce ketogenesis to facilitate autophagy activation. Consequently, the macronutrient composition of the regimen was not the primary focus of the study, as the patients' nutritional needs were largely met by endogenous sources through the breakdown of fat and protein. Conversely, some patients in the standard nutrition group (>400 kcal/day) were allowed to consume sugary drinks like juices and sports drinks in addition to their intravenous fluids, making it difficult to precisely measure their total caloric intake. Therefore, a maximum caloric intake for the standard nutrition group was not evaluated, as the primary objective of this study was to evaluate the impact of caloric restriction.

Prior to the implementation of caloric restriction, attending physicians assessed potential contraindications, including inborn errors of metabolism, such as pyruvate carboxylase deficiency, carnitine deficiency, pyruvate dehydrogenase deficiency, and porphyria, based on clinical history and laboratory data. In addition, patients with poorly controlled diabetes mellitus were screened to ensure patient safety.

In the calorie-restricted group, electrolyte and mineral balance were carefully monitored to prevent imbalances potentially caused by low caloric intake. Blood glucose levels were measured thrice per day during caloric restriction and seven times per day (before and after meals and before bedtime) after oral intake was resumed. To prevent refeeding syndrome, regular blood tests were performed to monitor serum electrolyte levels and metabolic status, ensuring appropriate nutritional management and timely intervention if abnormalities were detected.

**Figure 1:**
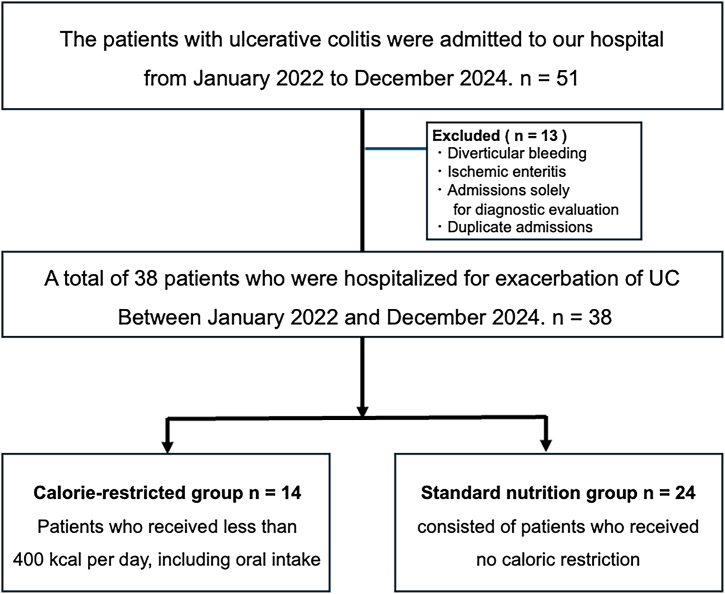
Flow diagram of patient selection and study group classification in the retrospective cohort. Patients hospitalized with ulcerative colitis between January 2022 and December 2024 were screened (n = 51). After excluding patients with diverticular bleeding, ischemic enteritis, admissions solely for diagnostic purposes, and duplicate admissions, 38 patients were included in the analysis. Patients were categorized into two groups based on caloric intake during hospitalization: calorie-restricted group (≤400 kcal/d, n = 14) and standard nutrition group (>400 kcal/d, n = 24).

Figure 1 shows a flow diagram of patient selection and group classification. Urinary ketone body testing was performed using a dipstick (“Uro-Paper α III,” Eiken Chemical Co, Ltd). The results were recorded by a clinical laboratory technologist in the electronic medical record. Urinary ketone bodies have been utilized as an accessible and cost-effective indicator of metabolic ketosis, a state often observed in conjunction with nutrient deprivation-induced autophagy.

However, it is crucial to acknowledge that the detection of urinary ketones primarily reflects a state of ketosis and overall catabolic metabolism and thus does not provide direct or specific evidence of autophagy activation. Therefore, the presence of urinary ketones in this study should be interpreted as an indirect suggestion of autophagy induction, and this limitation is explicitly recognized to avoid overinterpretation of the mechanistic findings.

All patients in the calorie-restricted group underwent urinary ketone testing, whereas in the standard nutrition group, testing was not routinely performed because of the retrospective nature of this study and the clinical discretion of the attending physicians. The patients underwent continuous monitoring of nutritional status, inflammatory markers, and clinical responses during hospitalization. Adverse events, including hypoglycemia, electrolyte imbalance, hepatic dysfunction (defined as alanine aminotransferase > 30 U/L), catheter-related infections, thromboembolic events, and fever, were systematically recorded. Documentation was based on the standard criteria used in UC clinical trials, and adverse events contributing to prolonged hospitalization were identified through an electronic medical record review.

### Statistical Analyses

Descriptive statistics were used to summarize the baseline patient characteristics. Continuous variables are expressed as means ± standard deviations. Given the small sample size, appropriate statistical methods were used to ensure the robustness of the results.

For between-group comparisons, the normality of continuous variables was assessed using the Shapiro–Wilk test. As both groups followed a normal distribution, equality of variance was tested using Levene’s test. Welch’s *t*-test was used because an equal variance was not confirmed. For continuous variables that did not follow a normal distribution, the Mann–Whitney U test was used. Categorical variables are presented as frequencies and percentages, and Fisher’s exact test was used for analysis.

Univariate and multivariate logistic regression analyses were performed to identify factors related to clinical remission. In the univariate analysis, logistic regression was conducted, and variables with a *P* value <.1 (sex and caloric restriction) were considered for further analysis. A multivariate logistic regression model was constructed using these variables, along with clinically relevant factors for UC outcomes, including PMS, MES, CRP, serum Alb, and previous biologic therapy. Odds ratios (ORs) with 95% confidence intervals (95% CIs) were calculated for the selected variables to determine their independent association with clinical remission.

All statistical analyses were performed using R software (version 4.5.1). The revised multivariate analysis was conducted with this updated version. A *P* value of < 0.05 was considered statistically significant.

### Ethics

This retrospective cohort study was conducted in accordance with the ethical principles of the Declaration of Helsinki (latest revision) and Guidelines for Medical Research Involving Human Subjects issued by the Japanese Ministry of Health, Labour and Welfare. The study protocol was reviewed and approved by the Ethics Committee of the Kindai University Hospital (approval no. R06-116).

Given the retrospective nature of this study, the requirement for written informed consent was waived by the Ethics Committee of Kindai University Hospital. Patient data were deidentified and anonymized before analysis to ensure confidentiality. Clinical data, including patient demographics, medication history, disease activity, and laboratory findings, were extracted and analyzed exclusively from electronic medical records.

## Results

### Patient Characteristics

A total of 38 hospitalized patients with UC were included in the analysis. Of them, 14 were assigned to the calorie-restricted group and 24 to the standard nutrition group. A representative case is presented in the Supplementary Case report to illustrate the treatment protocol in more detail. As shown in Table 1, baseline characteristics, including age, body mass index (BMI), disease duration, disease phenotype, fasting duration, PMS, MES, CRP level, Alb level, hemoglobin level, body temperature, heart rate at admission, smoking history, and treatment history (prior corticosteroid use or prior biologic therapy), did not differ significantly between the two groups.

**Table1:** Patient Characteristics

Characteristics	Calorie-restricted group n = 14	Standard nutrition group n = 24	*P* value
Age (mean ± SD)	46.6 ± 23.3	52.5 ± 21.3	.431
Sex (male: female)	Male: 11 female: 3	Male: 8 female: 16	.017^a^
Body mass index (BMI), mean	20.5 ± 4.7	20.0 ± 3.3	.65
Disease duration (mo), mean	66.9 ± 69	69.3 ± 120.8	.95
Disease type	Pancolitis: 13 Left-sided: 1	Pancolitis: 20 Left-sided: 4	.64
Duration of fasting (d)	6.6 ± 2.0	8.0 ± 5.7	.41
Partial Mayo score at admission, mean	7.1 ± 1.8	6.6 ± 1.8	.46
Mayo endoscopic subscore (MES), mean	2.50 ± 0.52	2.25 ± 0.61	.21
Full Mayo score at admission	9.57 ± 1.9	8.88 ± 2.0	.46
C-reactive protein (CRP) at admission (mean ± SD)	4.6 ± 5.1	3.6 ± 4.7	.55
Serum albumin at admission	3.50 ± 0.55	3.38 ± 0.54	.50
Body temperature at admission, mean	36.9 ± 0.72	37.0 ± 0.47	.60
Heart rate at admission, mean	81.0 ± 11.5	87.3 ± 17.3	.24
Hb (g/dL)	11.5 ± 2.2	11.1 ± 2.24	.64
Smoking history	5/14	8/24	n.s
Treatment history			
History of corticosteroid use	6/14	10/24	n.s
Biologic-naïve	10/14	18/24	n.s
History of biologic therapy	4/10	6/24	n.s
Anti-TNFα	2		
Vedolizumab	0	2	
JAK (TOF)	1		
Carotegrast methyl		1	
Anti-TNFα/Vedolizumab/UST	1		
JAK(FIL)/Anti-TNFα		1	
ADA/VED/UST/TAC/Mirikizumab		1	
Vedolizumab/UST		1	
Use of high-calorie parenteral nutrition	0/14	6/24	.07

∗*P* < .05.

Values are presented as mean ± standard deviation (SD) or number of patients, unless otherwise indicated.

*P* values were calculated using the Student’s *t*-test or Fisher’s exact test as appropriate.

“n.s.” indicates not significant (*P* ≥ .05).

ADA, adalimumab; anti-TNFα, anti–tumor necrosis factor alpha agent; FIL, filgotinib; Hb, hemoglobin; JAK, Janus kinase inhibitor; TAC, tacrolimus; TOF, tofacitinib; UST, ustekinumab; VED, vedolizumab.

a Indicates statistical significance (*P* < .05). ∗∗indicates statistical significance (*P* < .01).

In contrast, the proportion of female patients was significantly higher in the standard nutrition group than in the calorie-restricted group (*P* < .05). In addition, none of the patients in the calorie-restricted group required a transition to high-calorie PN during hospitalization, whereas 6 of the 24 patients in the standard nutrition group did.

### Primary Outcome: Clinical Remission at Day 14

**Figure 2:**
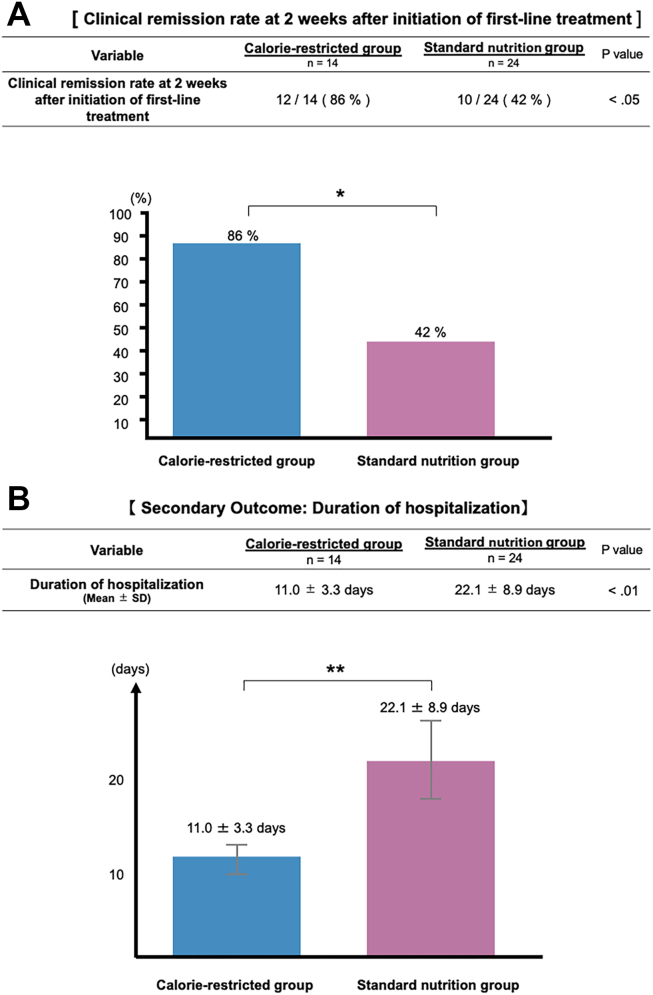
(A) Clinical remission rate at 2 weeks after initiation of first-line treatment. The clinical remission rate on day 14 after the initiation of first-line induction therapy was significantly higher in the calorie-restricted group than in the standard nutrition group (86% [12/14] vs 42% [10/24]). ∗*P* < .05 (Fisher's exact test). (B) Secondary outcome: duration of hospitalization. The mean duration of hospitalization was significantly shorter in the calorie-restricted group than in the standard nutrition group (11.0 ± 3.3 days vs 22.1 ± 8.9 days). ∗∗*P* < .01 (Mann-Whitney U test).

The clinical remission rate on day 14 after the initiation of first-line induction therapy was significantly higher in the calorie-restricted group than in the standard nutrition group (86% [12/14] vs 42% [10/24], *P* < .05, Table 2, Figure 2A). Statistical significance was confirmed using Fisher’s exact test.

**Table 2:** Treatments Administered During Hospitalization

Type of treatment	Calorie-restricted group n = 14	Standard nutrition group n = 24
Corticosteroids	11	14
Filgotinib	2	2
Tofacitinib	1	0
Upadacitinib		1
Adalimumab		1
Golimumab		1
Tacrolimus		1
Carotegrast methyl		2
Mirikizumab		1
Discontinuation of 5-ASA		1

Type of treatment	Calorie-restricted group n = 2 (14)	Standard nutrition group n = 9 (24)
Second-line medications		
Corticosteroids		1
Filgotinib	1	
Upadacitinib	1	3
Tofacitinib		2
Golimumab		1
Ustekinumab		1
Azathioprine		1

Type of treatment	Calorie-restricted group n = 0 (14)	Standard nutrition group n = 1 (24)
Third-line medications		
Corticosteroids		1

Values indicate the number of patients who received each treatment during hospitalization.

Induction therapy was administered at the attending physician’s discretion. In the calorie-restricted group, 11 patients received corticosteroids, two received filgotinib, and one received tofacitinib. Among those treated with corticosteroids, two failed to achieve remission: one required a switch to upadacitinib owing to psychiatric symptoms and insomnia, and the other experienced symptom recurrence within 2 weeks and required switching to filgotinib. Remission was successfully achieved with second-line therapy in both cases, and none of the patients required third-line therapy.

In the standard nutrition group, the initial therapies included corticosteroids (n = 14), filgotinib (n = 2), carotegrast methyl (n = 2), upadacitinib (n = 1), adalimumab (n = 1), golimumab (n = 1), tacrolimus (n = 1), and mirikizumab (n = 1). One patient did not receive induction therapy because symptom exacerbation was suspected to be caused by 5-ASA allergy.

**Figure 3:**
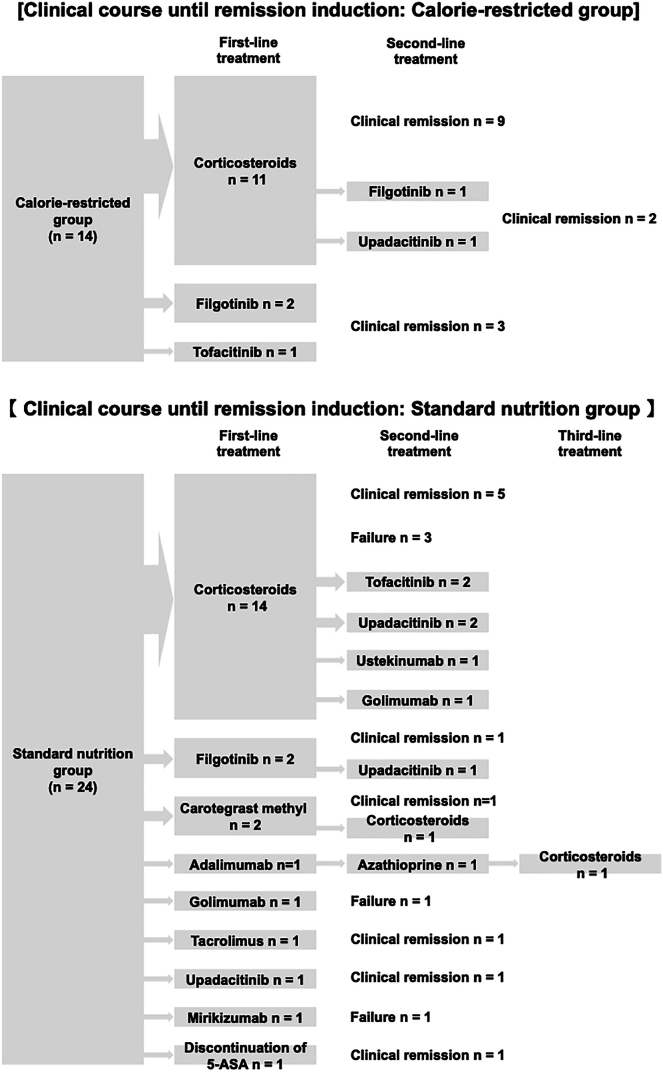
Clinical course until remission idard nutrition group.

Second-line treatment was administered in nine patients, including upadacitinib (n = 3), tofacitinib (n = 2), corticosteroids (n = 1), golimumab (n = 1), ustekinumab (n = 1), and azathioprine (n = 1). One patient subsequently required third-line corticosteroid therapy (Table 2, Figure 3).

### Univariate and Multivariate Analyses

Univariate and multivariate logistic regression analyses were performed to identify factors related to clinical remission (Table 3). In the univariate analysis, caloric restriction (*P* = .014; OR, 8.4; 95% CI, 1.53–46.111) and sex (*P* = .053; OR, 0.2597; 95% CI, 0.066–1.02) were considered relevant variables; these were included in the multivariate model. To address concerns regarding confounding factors, an expanded multivariate model was constructed that also included baseline disease severity (PMS and MES), nutritional status (serum Alb), previous biotherapy exposure, and CRP.

**Table3:** Logistic Regression Analysis of Factors Associated With Clinical Remission at Day 14 Following First-Line Therapy

Variable	Univariate analysis				Multivariate analysis			
	*P* value	OR	95% CI		*P* value	OR	95% CI	
Age	.833	1.0032	0.974	1.034				
BMI	.747	1.0256	0.879	1.196				
Duration of disease	.742	0.999	0.993	1.005				
PMS	.905	1.0229	0.706	1.482	.248	0.711	0.400	1.267
MES	.386	0.6012	0.19	1.901	.238	0.261	0.028	2.424
CRP	.636	1.0342	0.9	1.189	.041^a^	1.382	1.013	1.884
Alb	.117	2.9209	0.763	11.175	.064	13.573	0.861	214.0
Hb	.67	1.0669	0.792	1.437				
Duration of fasting	.138	0.8839	0.751	1.041				
Sex Male Female	.053	1 0.2597	0.066	1.02	.110	0.151	0.015	1.534
Previous biotherapy < 2 ≧ 2	.393	1 0.393	0.063	2.958	.093	0.027	0.0002	1.832
Caloric restriction yes no	.014^a^	8.4 1	1.53	46.111	.025^a^	20.666	1.471	290.4

“Previous biotherapy” refers to the number of biological agents used prior to hospitalization.

“Caloric restriction” refers to implementation of the calorie-restricted nutritional intervention during hospitalization.

Sex and previous biotherapy were treated as categorical variables, with female and <2 biologics as reference groups, respectively.

Hb, hemoglobin.

a Indicates statistical significance (*P* < .05).

In this expanded model, caloric restriction remained a statistically significant independent predictor of clinical remission (*P* = .025; OR, 20.666; 95% CI, 1.471–290.4). CRP was also a significant predictor of remission (*P* = .041; OR, 1.382; 95% CI, 1.013–1.884). In contrast, sex was not a significant predictor of remission (*P* = .110; OR, 0.151; 95% CI, 0.015–1.534).

### Assessment of Autophagy Activation

Urinary ketone bodies were evaluated as indirect biomarkers of autophagy activation. In the calorie-restricted group, routine testing confirmed ketonuria in all patients within 1–3 days of hospital admission. In contrast, routine testing was not performed in the standard nutrition group because it was left to the discretion of the attending physician.

### Secondary Outcomes: Duration of Hospitalization and Adverse Events

The mean duration of hospitalization was significantly shorter in the calorie-restricted group than in the standard nutrition group (11.0 ± 3.3 days vs 22.1 ± 8.9 days; *P* < .01; Figure 2B).

No serious adverse events (SAEs) were observed in the calorie-restricted group. In contrast, four patients in the standard nutrition group developed SAEs, including catheter-related infections (n = 2), *Clostridium difficile* infection (n = 2), and myocarditis (n = 1). A total of five SAEs occurred, as one patient experienced both a catheter-related infection and myocarditis. Mild adverse events observed in the calorie-restricted group included transient nausea and abdominal bloating related to hypoglycemia (n = 2) as well as insomnia and mood disturbances attributed to corticosteroid use. None of these events led to prolonged hospitalization.

Liver dysfunction, defined as serum alanine aminotransferase ≥ 30 U, was significantly more frequent in the standard nutrition group than in the calorie-restricted group (50% [12/24] vs 14% [2/14]; *P* < .05; Table 4). Electrolyte and mineral imbalances were appropriately managed by routine laboratory monitoring in both groups. No cases of refeeding syndrome were observed following the resumption of oral intake.

**Table 4:** Adverse events

Adverse events	Calorie-restricted group n = 14	Standard nutrition group n = 24	*P* value
Serious adverse events (SAEs)	0	Myocarditis 1 catheter-related infections 2 Clostridioides difficile colitis 2	.137
Nonserious adverse events	Nausea and epigastric discomfort associated with hypoglycemia 2 Insomnia and mood disturbance associated with prednisolone 1 total 3 cases	Cystitis 1 Nausea and epigastric discomfort Fever of unknown origin 3 Delirium 1 Eczematous rash 1 Total 6 cases	.684
Hepatic dysfunction (ALT ≥ 30 IU/L)	2/14	12/24 (among the 6 patients who received TPN, 5 developed hepatic dysfunction.)	.039^a^

^∗^*P* < .05.

Values represent the number of events observed in each group.

Serious adverse events (SAEs) included myocarditis, catheter-related infections, and *Clostridioides difficile* colitis.

Nonserious adverse events included symptoms such as nausea and epigastric discomfort (likely related to hypoglycemia), insomnia and mood disturbance (associated with prednisolone), fever of unknown origin, delirium, cystitis, and eczematous rash.

Hepatic dysfunction was defined as alanine aminotransferase (ALT) ≥ 30 IU/L.

*P* values were calculated using Fisher’s exact test.

a Indicates statistical significance (*P* < .05).

## Discussion

### Impact of Caloric Restriction on Clinical Outcomes in Hospitalized Patients With UC

This study demonstrated that despite no significant differences in fasting duration between groups, strict caloric restriction was linked to a significant reduction in hospital stay (calorie-restricted group: 11.0 d vs standard nutrition group: 22.1 d, *P* < .01) and improved success rate of first-line remission induction. Rather than aiming for simple bowel rest, caloric restriction was designed to activate autophagy, thereby inducing metabolic changes that facilitate remission induction and subsequently shorten the hospital stay.

Two key factors likely contributed to these findings. First, the significantly higher remission induction rate in the calorie-restricted group led to earlier symptom improvement. Second, the absence of severe adverse events in the calorie-restricted group, such as catheter-related infections and myocarditis, which were observed in the standard nutrition group, may have contributed to better clinical outcomes. Notably, none of the patients in the calorie-restricted group required central venous catheter (CVC) placement or high-caloric PN, whereas 6 of 24 (25%) patients in the standard nutrition group underwent CVC placement, potentially increasing the risk of infections.

Adverse events in the calorie-restricted group were mild and transient, including nausea, vomiting, and hypoglycemia. These symptoms are well-documented as part of the “keto flu,” which occurs during the metabolic transition from glycogen depletion to ketosis. This transition is characterized by hypoglycemia, nausea, vomiting, and dyslipidemia.^13^, ^14^, ^15^ Although gastrointestinal symptoms, such as nausea, vomiting, diarrhea, and constipation, may occur during the metabolic shift toward ketone body utilization, these effects are transient and generally do not require specific interventions.^6^, ^7^, ^8^

Conversely, among the 6 patients in the standard nutrition group who received high-caloric PN, 3 developed liver dysfunction, including one case of myocarditis (later diagnosed as infectious endocarditis with cerebral infarction) and one case of catheter-related infection, both of which were classified as severe adverse events.

These findings align with those of previous studies, suggesting that total PN (TPN) increases the risk of complications in hospitalized patients with UC. In a randomized controlled trial by González-Huix et al., involving 42 hospitalized patients with severe UC, there was no significant difference in remission or surgery avoidance rates between TPN and EN groups, but the incidence rate of adverse events was significantly higher in the TPN group (35%) than in the EN group (9%).^16^

CVC-related infections were frequently reported in the TPN group, reinforcing the notion that TPN use may elevate the risk of complications in patients with UC. The findings of this study support the idea that avoiding unnecessary CVC placement may be beneficial for hospitalized patients with UC.

### Univariate and Multivariate Analyses

Univariate and multivariate logistic regression analyses were performed to identify factors associated with clinical remission (Table 3). In the univariate analysis, caloric restriction (*P* = .014; OR, 8.400; 95% CI, 1.53–46.111) and sex (*P* = .053; OR, 0.259; 95% CI, 0.066–1.02) were considered relevant variables and these were included in the multivariate model. To address concerns regarding confounding factors, an expanded multivariate model was constructed that also included baseline disease severity (PMS and MES), nutritional status (serum Alb), previous biotherapy exposure, and CRP.

In this expanded model, caloric restriction remained a statistically significant independent predictor of clinical remission (*P* = .025; OR, 20.666; 95% CI, 1.471–290.4). CRP was also a significant predictor of remission, but the direction of this association appears paradoxical (*P* = .041; OR, 1.382; 95% CI, 1.013–1.884). This finding may be attributed to the complex interplay and potential collinearity among other disease activity markers (e.g., PMS) included in the model. In contrast, sex was not a significant predictor of remission (*P* = .110; OR, 0.151; 95% CI, 0.015–1.534).

### Potential Sex-Based Differences in Response to Caloric Restriction

One noteworthy limitation of this study was the significant difference in male-to-female ratio between groups, which warrants a cautious interpretation of the results. As this was a retrospective study, the observed sex distribution imbalance can be attributed to chance.

A closer analysis of female patients in the calorie-restricted group revealed that one out of three failed first-line remission induction, whereas another experienced disease exacerbation within 4 weeks. In contrast, all male patients in the calorie-restricted group successfully achieved remission induction, with no relapses within 4 weeks.

However, due to the small subgroup size, especially with only three females in the calorie-restricted group, any potential sex-based differences in response to caloric restriction must be interpreted with extreme caution. The observed trend should not be overstated, and further studies with larger, balanced cohorts are necessary to validate these preliminary findings.

The potential for sex-specific responses can be attributed to physiological metabolic differences.^17^^,^^18^ Metabolic responses in women are influenced by menstrual cycles, body fat distribution, and hormonal regulation, all of which differ significantly from those in males. Women generally have lower fatty acid oxidation rates than men, resulting in reduced ketone body production during fasting. Additionally, estrogen modulates lipid metabolism and insulin sensitivity, potentially leading to differential fasting responses between sexes.^10^^,^^11^

Furthermore, women with low BMI may have limited fat reserves, restricting the availability of fatty acids as an energy substrate, thereby reducing ketone body production during fasting. In individuals with extremely low BMI, hepatic ketone body synthesis is often insufficient to compensate for energy deficits and increases susceptibility to energy depletion and metabolic stress.^12^

These physiological differences could potentially explain the observed trend, but again, this remains a hypothesis that requires robust validation in future studies.

### Caloric Restriction as a Novel Nutritional Strategy for UC

This study explored the efficacy of caloric restriction (≤400 kcal/d) aimed at autophagy induction, rather than simple bowel rest, in hospitalized patients with acute UC exacerbation. Compared with high-caloric PN, caloric restriction was associated with a shorter hospital stay and a higher remission induction rate.

Traditional nutritional management strategies for UC have not demonstrated a significant impact on disease activity, and early initiation of EN is generally recommended.^4^^,^^5^ However, previous studies have focused on bowel rest as the primary objective, whereas the approach in this study specifically aimed to activate autophagy through low-caloric intake.

Urinary ketone body positivity in the calorie-restricted group suggests that autophagy activation was successfully induced, potentially contributing to disease improvement. Many nutritional studies have overlooked the adverse effects of excessive caloric intake, such as hyperglycemia and hyperinsulinemia, both of which may exacerbate inflammation.^6^^,^^7^ Given that the EPaNIC trial demonstrated that high-caloric nutrition inhibited autophagy, impairing cellular repair and inflammation resolution, the present findings provide new clinical evidence supporting this theory.

Although mild transient adverse events (nausea, hypoglycemia) were observed in the calorie-restricted group, no SAEs occurred. Conversely, in the standard nutrition group, complications, such as catheter-related infections and thrombosis, were observed, suggesting a potential safety advantage of caloric restriction therapy.

### Autophagy and Immune Modulation in UC and Other Autoimmune Diseases

Although limited to case reports, previous studies have suggested that caloric restriction and intermittent fasting may reduce systemic inflammatory markers, including interleukin-6 and CRP, in patients with UC.^19^ Similar findings have been reported in other autoimmune diseases treated with biologics, such as rheumatoid arthritis (RA) and psoriasis. For RA, fasting was found to significantly improve subjective symptoms, although the effects were transient.^20^ One study showed that patients with RA undergoing fasting exhibited rapid symptom improvement within the first week, whereas symptom reduction in the control group was delayed until 6 weeks postintervention.^21^ In psoriasis, clinical trials investigating the combination of immunosuppressive agents or biologics with low-calorie diets have demonstrated greater improvement in skin lesions than pharmacological treatment alone.^22^

Autophagy activation is recognized as a crucial mechanism for cellular homeostasis and inflammation control.^8^ IBDs, including UC and Crohn’s disease, are characterized by autophagy pathways, with autophagy-related genes (*ATG16L1, immunity-related GTPase M*) identified as the susceptibility loci.

Recent studies have provided further insights into the therapeutic potential of autophagy modulation in IBD. For example, a previous study demonstrated that a fasting-mimicking diet in a murine colitis model led to increased intestinal stem cell proliferation, beneficial microbiota shifts, and a marked reduction in inflammation and tissue damage.^23^

## Study Limitations and Future Perspectives

This study has several limitations that warrant careful consideration. First, the retrospective, single-center design with a small sample size may limit the generalizability of our findings. Importantly, the nonrandomized allocation of patients to the two groups, which was primarily driven by the discretion of the attending physicians, introduced a significant potential for selection bias. For example, unmeasured factors such as initial disease severity, response to prior treatments, or patient characteristics may have influenced the physician's decision to implement the caloric restriction protocol. While baseline characteristics were comparable, we cannot exclude the possibility of unmeasured confounding variables.

Second, as noted previously, the use of urinary ketone bodies as an indirect biomarker for autophagy, rather than a direct, quantitative measure, is a key mechanistic limitation. To address these limitations and validate our hypothesis-generating findings, future research must involve prospective, randomized, multicenter trials that incorporate direct autophagy biomarkers and control for potential confounders.

## Conclusion

In hospitalized patients with ulcerative colitis, a carefully monitored caloric restriction regimen (< 400 kcal/day) was associated with a higher rate of clinical remission and a shorter duration of hospitalization compared with standard nutritional management. These findings suggest that caloric restriction aimed at promoting autophagy may offer a safe, feasible, and cost-effective approach in the acute management of UC. Further prospective, multicenter studies are required to validate these observations and clarify the underlying mechanisms.

## Figures and Tables

**Supplementary Case Report:**